# A Straightforward Method to Prepare MOF-Based Membranes via Direct Seeding of MOF-Polymer Hybrid Nanoparticles

**DOI:** 10.3390/membranes13010065

**Published:** 2023-01-04

**Authors:** Mingyuan Fang, Martin Drobek, Didier Cot, Carmen Montoro, Mona Semsarilar

**Affiliations:** 1 Institut Européen des Membranes, IEM UMR 5635, University of Montpellier, CNRS, ENSCM, 34095 Montpellier, France; 2 Inorganic Chemistry Department, Universidad Autónoma de Madrid, 28049 Madrid, Spain

**Keywords:** metal-organic frameworks, UiO-66, nanoparticles, seeding, secondary growth, membranes

## Abstract

Metal Organic Frameworks (MOFs) present high surface areas, various pore topology as well as good stabilities. The functionalities and porosity can be tuned by using different linkers with various functional groups and a wide range of linker lengths. These properties make them good candidates in membrane separation applications. In this work, we propose a simple UiO-66 MOF-based membrane fabrication method following two steps. First, the α-alumina tubular membrane support was dip-coated with MOF-polymer hybrid nanoparticles (NPs). These NPs were prepared via one-pot synthesis by adding poly (methacrylic acid)-*b*-poly (methyl methacrylate) (PMAA-*b*-PMMA) NPs to the classical acetic acid-modulated UiO-66 or UiO-66-NH_2_ synthesis formulation. Second, secondary membrane growth was applied to give rise to a continuous and homogeneous crystalline MOF membrane layer. The gas permeances (He, N_2_, CO_2_ and SF_6_) tests confirmed high membrane permeability with no macro-defects. The as-prepared membranes that were used for dye separation (Rhodamine B) showed relatively good separation capacity.

## 1. Introduction

Metal Organic Frameworks (MOFs) are well-known crystalline porous materials based on metal ions or clusters coordinated to organic linkers following the principle of reticular chemistry [1,2]. MOFs can be found in the medical, environmental, energy, agricultural, textile, and food industries [3,4,5,6]. They are characterized by high surface areas, tunable pore size and functionalities, as well as physical–chemical stabilities. Moreover, these interesting properties make them attractive candidates in membrane separation applications such as gas purification, water treatment and pervaporation [7,8,9,10].

MOF-based membranes usually are prepared onto inorganic, or polymer supports due to their poor mechanical properties. In this sense, many strategies have been developed considering bottom-up or top-down approaches [11]. For the first case, the direct or in situ growth of MOFs on a porous support via solvothermal or microwave-assisted synthesis is considered one of the most classic strategies [12,13,14,15]. Alternative supports such as polydopamine-functionalized stainless-steel nets have also been used for the preparation of MOF-based membranes showing high quality [16]. Although numerous MOF-based membranes were prepared following this strategy, special designed synthesis conditions or modified supports are often required [17]. In comparison with direct growth, the application of secondary or reactive growth in general represents a more attractive strategy since it ensures the synthesis of thin, homogeneous, and defect-free selective layers on the supports [18]. This approach requires a suitable seeding of the supports either with the specific precursors (reactive seeding) or nano-seeds of the same material composition as the final membrane layer (classical seeding) [19,20]. Whatever the selected method, the seeding should be regular, covering the entire active surface of the support, for ensuring a homogeneous growth of continuous selective membrane layers. In the area of MOF-based membrane synthesis using reactive seeding, different strategies have been considered applying either the deposition of organic ligands or metal oxide on the membrane supports [21,22]. For example, Du et al. [23] immobilized 2-aminoterephthalic acid on an α-alumina support targeting the preparation of UiO-66-NH_2_ membrane. Drobek et al. [24] deposited a thin film of ZnO by Atomic Layer Deposition which served as metal source (Zn^+2^) for solvothermal growth of ZIF-8 membrane layers for hydrogen separation. These reactive seeding methods led to a good control over the quality of the as-seeded layers. However, most of these methods require complex deposition techniques. In contrast, classical seeding approaches do not require any sophisticated deposition protocols and could be easily achieved by dip-coating or slip-casting from the seed suspensions. For example, Lai et al. [25] reduced ZIF-69 seeds to sub-micro size by using zinc nitrate instead of zinc acetate as synthetic precursor. The as-prepared ZIF-69 seeds were then coated on porous α-alumina discs before the secondary growth. Caro et al. [26] prepared 300 nm octahedral UiO-66 crystal seeds which were used for the secondary growth of a highly oriented UiO-66 membrane. It should be emphasized that the classical seeding is simple to perform but small MOF particles should be synthetized for preparing stable seed suspensions.

Recently, a new top-down approach which combines both the obtention of mixed matrix membranes and the membrane-seeding strategies has been studied via deposition of a homogenous dispersion containing polymer matrix with MOF crystals on the surface of the membrane supports. For example, Li et al. [27,28] have mixed polyethyleneimine with previously prepared ZIF-7 powder to obtain a homogeneous dispersion of MOF seeds in the polymer. Obviously, such a polymer-assisted seeding approach requires the preparation of MOF crystals with an optimal size and a suitable polymer at defined concertation.

Apart from the membrane preparation problems, mainly derived from obtaining a dense polycrystalline layer on supports, the stability problems of a MOF also have to be considered. Among all the existing MOFs, that one containing high-valence Zr^4+^ cations, such as the well-known UiO (UiO from Universitet i Oslo) family MOFs [29], show remarkable stability properties in terms of thermal, chemical and mechanical stability. Moreover, this family of MOFs could also be easily synthesized on a large scale [30,31].

In this work, we propose a simple and direct seeding method to prepare MOF-based membranes by using our previously reported UiO-polymer hybrid nanoparticles (NPs) based on poly (methacrylic acid)-*b*-poly (methyl methacrylate) (PMAA-*b*-PMMA) NPs totally different from those already reported [32,33]. We have focused on UiO-66 and UiO-66-NH_2_ MOFs as they are highly stable in water which is an essential requirement for practical applications such as water treatment [34]. Several advantages could be highlighted using this approach. Firstly, the facile synthesis of UiO-PMAA-*b*-PMMA NPs via one-pot synthesis by adding PMAA-*b*-PMMA NPs to the classical acetic acid-modulated UiO synthesis formulation. Secondly, the resulting, highly stable colloidal solution that allows for direct seeding with no extra treatments feasible. Thirdly, the seeding quantity and its density could be controlled by simply managing the concentration of the UiO-PMAA-*b*-PMMA colloidal solution. In this case, two types of UiO-polymer hybrid NPs (UiO-PMAA-*b*-PMMA and UiO-NH_2_-PMAA-*b*-PMMA) were prepared and deposited on α-alumina supports via dip-coating. The UiO-PMAA-*b*-PMMA NPs were covered in our previous report [32] while the amine-functionalized NPs (UiO-NH_2_-PMAA-*b*-PMMA) are reported here for the first time. The NP-seeded supports were then subjected to solvothermal treatments (secondary membrane growth) to grow a continuous crystalline layer on them. The prepared MOF-based membranes were analyzed through gas permeation tests and dye separation experiment.

## 2. Materials and Methods

### 2.1. Materials

Methacrylic acid (MAA) 99.0% purity, 4-cyano-4-(phenylcarbonothioylthio)pentanoic acid > 97.0% purity, 4,4′-azobis(4-cyanovaleric acid) (ACVA) 98.0% purity, methyl methacrylate (MMA) 99.0% purity, 2,2′-azobis(2-methylpropionitrile) (AIBN) 98.0% purity, ethylene glycol dimethacrylate (EGDMA) 98.0% purity, (trimethylsilyl)diazomethane solution 2.0 M in diethyl ether, zirconium (IV) chloride (ZrCl_4_) ≥ 99.5% purity, 2-aminoterephthalic acid 99.0% purity and Rhodamine B (RhB) ≥ 95% purity were supplied by Sigma-Aldrich (San Luis, MO, USA). Terephthalic acid ≥ 98.0% purity was supplied by Alfa Aesar (Kandel, Germany). Solvents were acquired from VWR and Fisher Scientific (Radnor, PA, USA and Waltham, MA, USA, respectively). All other reagents were used as received.

The α-alumina tubular membrane supports (Ø_pore_ = 200 nm, OD/ID = 10/7 mm) were purchased from PALL-EXEKIA (Bazet, France) and cut to 5 cm long pieces. The membranes have an asymmetric structure with three different porosities: 0.2 µm pores sizes for the inner layer (thickness about 10 µm), 0.8 µm for the intermediate layer (thickness about 20 µm) and 10 µm for the external layer (Figure S1 (Supplementary Materials)). To avoid gas leakage, both membrane extremities were sealed with a commercial enamel (1 cm inside and outside).

### 2.2. Method

#### 2.2.1. The Synthesis of Poly(Methacrylic Acid) (PMAA) and PISA Synthesis of Poly(Methacrylic Acid)–b-Poly(Methyl Methacrylate) Diblock Copolymer (PMAA-b-PMMA) NPs

PMAA and PMAA-*b*-PMMA NPs was completed following a previously reported method [32].

#### 2.2.2. Synthesis of UiO-PMAA-b-PMMA and UiO-NH_2_-PMAA-b-PMMA NPs

UiO-PMAA-*b*-PMMA colloids were prepared using a similar strategy that we have already reported [32]. Briefly, PMAA-*b*-PMMA NPs 20% *w*/*w* in ethanol (20% molar ratio of carboxylic acid function of PMAA-*b*-PMMA to zirconium; see details in Table S1) were dispersed in 2 mL of N,N-dimethylformamide (DMF) and stirred for 1 h. ZrCl_4_ (0.5 mmol, 106.5 mg) and terephthalic acid (0.5 mmol, 83 mg) for the UiO-PMAA-*b*-PMMA or 2-aminoterephthalic acid (0.5 mmol, 90.5 mg) for the UiO-NH_2_-PMAA-*b*-PMMA were dissolved separately in 3 mL DMF by sonication for about 10 min. ZrCl_4_ solution was then mixed with PMAA-*b*-PMMA NPs and terephthalic acid or 2-aminoterephthalic acid as well as 0.5 mL of acetic acid were then added. The resulting mixture was sonicated for 2 min and transferred to a 20 mL cylindrical glass pressure vessel that was heated at 120 °C. After 20 h, light pink in the case of UiO-PMAA-*b*-PMMA and yellow in the case of UiO-NH_2_-PMAA-*b*-PMMA colloidal solutions of NPs in DMF were obtained. To obtain the dry powder of UiO-NH_2_-PMAA-*b*-PMMA for characterization, the yellow UiO-NH_2_-PMAA-*b*-PMMA solution was centrifuged at 4.4 K rpm for 20 min and washed with 2 × 10 mL of DMF. The washing and centrifugation cycles were repeated using ethanol (3 × 10 mL). The washed powders were then dried under vacuum at 80 °C for 8 h.

#### 2.2.3. Synthesis of UiO-66 and UiO-66-NH_2_

UiO-66 and UiO-66-NH_2_ were synthesized using the method previously reported by Behrens et al. [35] considering 30 equivalent of acetic acid (0.5 mL), 0.25 mmol (53.3 mg) of ZrCl_4_, 0.25 mmol (41.5 mg) of terephthalic acid for UiO-66 or 0.25 mmol (45.3 mg) of 2-aminoterephthalic acid for UiO-66-NH_2_ in 4 mL of DMF.

#### 2.2.4. Membranes’ Preparation

**Scheme 1:**
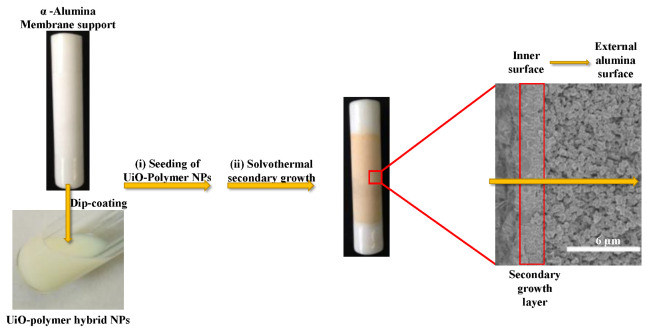
Scheme of the dip-coating and secondary growth procedure for UiO-66-NH_2_ membranes.

UiO-PMAA-*b*-PMMA and UiO-NH_2_-PMAA-*b*-PMMA membranes were prepared by the secondary growth method on the α-alumina tubular supports (Scheme 1). In a first step, UiO-PMAA-*b*-PMMA or UiO-NH_2_-PMAA-*b*-PMMA colloidal NPs were deposited following the dip-coating technique. For that, the α-alumina membranes were immersed in the corresponding colloidal solution for about 1 min, then slowly pulled out (1 cm min^−1^) and dried in a vertical position for 24 h (in air). The same coating procedure was repeated one more time, followed by the final support drying at 120 °C under vacuum for 24 h. The quantity of deposited material was monitored by the support weight increase after the dip-coating process. The optimal weight increase was in the range of 0.1 to 0.2 wt.% (around 10 mg) for the applied seed suspensions. The as-modified (seeded) supports were subjected to a secondary growth method under solvothermal conditions in a cylindrical glass pressure vessel. Typically, ZrCl_4_ (0.5 mmol, 116.5 mg), terephthalic acid (0.5 mmol, 83.1 mg) or 2-aminoterephthalic acid (0.5 mmol, 90.6 mg) were dissolved in 20 mL DMF by sonication. Then, 9 µL water was added into the solution following the recommended ration of Zr^4+^/BDC/H_2_O/DMF = 1:1:1:500 [15]. This solution was transferred to a cylindrical glass pressure vessel in which α-alumina tubular membrane supports were placed in vertical position and heated at 120 °C for 72 h. After cooling down the vessel, the obtained membranes were washed with 2 × 20 mL DMF and 3 × 20 mL ethanol and subsequently dried at room temperature in open air overnight and followed by vacuum drying at 80 °C for 12 h. The observed support weight increase was in the range of 0.3 to 0.5 wt.% (around 30 mg) for both as-synthetized UiO-66 or UiO-66-NH_2_ membranes grown on the α-alumina membrane support.

#### 2.2.5. Gas Permeation Experiments

Single gas permeation tests were performed at room temperature under different transmembrane pressures (*ΔP* = 0.25, 0.5 and 1 bar). Single gas flow across the membrane layer (6.6·10^−4^ m^2^ active surface area) was measured by a 0.05 L bubble flowmeter connected to the atmosphere. Permeation was evaluated with four pure gases (He, N_2_, CO_2_ and SF_6_). The membrane degassing (primary vacuum pump) was performed before and after each gas test. Single gas permeance (*P*) was calculated using the following equation:

(1) $$P=\frac{n}{A\cdot \Delta P\cdot t}$$

where *n* is the quantity of gas passing through the membrane (mol) and was calculated from the equation of ideal gas (R = 0.082 (L atm)·(mol K)^−1^, *n* = 2.05 × 10^−3^ mol). *ΔP* is the transmembrane pressure (Pa, 1 bar = 10^5^ Pa), *t* is time (s) and *A* is the membrane active surface area (m^2^).

The ideal (single) gas selectivity of gas *i* over gas *j* was calculated as a ratio of the corresponding single gas permeances, Equation (2):

(2) $$\alpha_{i/j}=\frac{P_{i}}{P_{j}}$$

Ideal gas selectivity was compared with that of Knudsen selectivity calculated according to Equation (3):

(3) $$K_{i/j}=\sqrt{\frac{M_{j}}{M_{i}}}$$

where *M* (g·mol^−1^) is the molar mass of gas *j* and *i*, respectively.

#### 2.2.6. Nanofiltration

The nanofiltration experiment was performed on the same membrane module after gas permeation tests. The membrane module was coupled to a compressed air line and a water reservoir. In a first step, the membrane was stabilized through distilled water infiltration for 2 h, increasing the pressure from 0.5 to 2 bar. Then, 2 bar pressure was kept for another 2 h and the water excess was removed by dabbing with paper towel. Dye separation experiment was carried out using 10 mL aqueous solution of RhB (~0.03 mM) at 1 bar. The volumetric flux (*J_v_*, L·h^−1^ m^−2^) and permeability (*L_P_*, L·h^−1^ m^−2^ bar^−1^) were calculated using the equations:

(4) $$J_{v}=\frac{V_{p}}{t\cdot S}$$

(5) $$L_{P}=\frac{J_{v}}{\Delta P}$$

where *V_p_* indicates the permeate volume (L); *t* the time for collection of permeate (h); *S* membrane area (m^2^) and *ΔP* the pressure drops through the membrane (bar). Dye rejection was estimated by Equation (6) as follows:

(6) $$D_{REJ}\left(\%\right)=\left(1-\frac{C_{p}}{C_{0}}\right)\cdot 100$$

Being *C_p_* and *C*_0_ the solute concentration in permeate and feed, respectively. These values were measured using a UV spectrophotometer (SHIMADZU UV-2401PC spectrophotometer, Kyoto, Japan) at 554 nm wavelength of light which corresponds to the maximum wavelength of RhB. The permeate was collected and analyzed at fractions of 2.5 mL.

### 2.3. Characterization

The surface morphologies of the materials were characterized by Scanning Electron Microscopy (SEM, Hitachi S4800, Tokyo, Japan) images obtained at 0.1−30 kV working voltage and Transmission Electron Microscopy (TEM, JEOL 1200 EXII, Peabody, MA, USA) images under working voltages up to 120 kV. The image analysis was performed using ImageJ software. The cross-sectional membrane samples were fractured mechanically. The crystalline structure of the samples was analyzed by Powder X-ray diffraction (PXRD) measurements at room temperature on a X’pert Pro diffractometer (PAN Analytical, Malvern, England) working in reflectance parallel beam/parallel slit alignment Bragg–Brentano geometry and equipped with a X’Celerator detector. The measurement employed Ni-filtered Cu Kα line focused radiation at 800 W (40 kV, 20 mA) power. Samples were observed using a 0.017° 2θ step scan from 5° to 50° with an exposure time of 60 s per step. Chemical composition of the samples was analyzed by Fourier-transform infrared (FT-IR, Thermo Nicolet iS50 FT-IR, Carlsbad, CA, USA) spectra performed in transmission mode. The porosity of the samples was determined by nitrogen adsorption isotherms measured at 77 K on a Micromeritics ASAP 2020 Plus Adsorption Analyzer (Unterschleissheim, Germany). Prior to measurement, powder samples were degassed at 373 K for 12 h.

## 3. Results

### 3.1. Synthesis and Characterization of MOF-Polymer Hybrid NPs

We have recently reported the synthesis of UiO-polymer hybrid NPs through the addition of core cross-linked PMAA-*b*-PMMA NPs to a classical formulation of UiO-66 synthesis [32]. As was demonstrated, the presence of multi-carboxylic acid groups on the surface of these well-defined spherical NPs leads their coordination with the metal ions and clusters to grow the MOF. It is important to highlight that the PXRD analysis of microcrystalline MOFs is crucial to demonstrate the maintenance of structures [36,37]. Thus, the resulting UiO-PMAA-*b*-PMMA NPs showed good crystallinity and porosity together with long-term colloidal stability. As stated, this approach can be easily applied for the synthesis of other carboxylic acid-based UiO MOFs with side functionalities. So, in this case, we have performed the same strategy for the synthesis of UiO-NH_2_ MOF in the presence of core cross-linked PMAA-*b*-PMMA NPs. Then, we obtained UiO-NH_2_-PMAA-*b*-PMMA NPs which also exhibited good colloidal stability and feature spherical shape with sharp angular edges and a particle size between 90 to 130 ± 10 nm as observed by TEM and SEM (Figure S2a,b,c). Moreover, they showed similar colloidal stability and no precipitation after being left unstirred for a month at ambient conditions. The details of the UiO-NH_2_-PMAA-*b*-PMMA NPs characterization (PXRD, FT-IR, Nitrogen adsorption) are shown in Figures S3–S5 (Supplementary Materials)).

### 3.2. Membranes Seeding with UiO-Polymer Hybrid NPs

**Figure 1:**
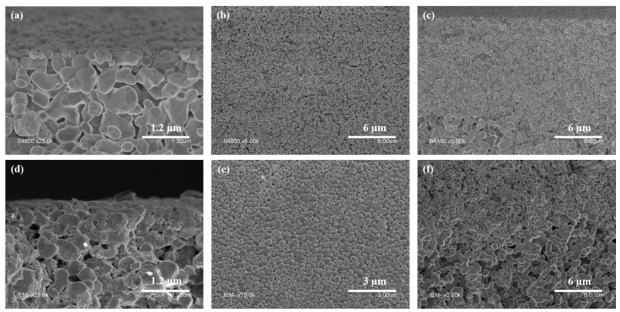
SEM images of (**a**) inner support layer cross-section, (**b**) inner support surface top view, (**c**) inner and intermediate support layer cross-section modified with UiO-PMAA-*b*-PMMA NPs; (**d**) inner support layer cross-section, (**e**) inner support surface top view, (**f**) inner and intermediate support layer cross-section modified with UiO-NH_2_-PMAA-*b*-PMMA.

In view of the good colloidal stabilities, both MOF-polymer hybrid NPs suspensions were used for the seeding of α-alumina membrane supports via dip-coating. The SEM images (Figure 1) showed that the depositions of both NPs were successful, specifically on the inner surface of the α-alumina membrane support. The thin deposited layers (Figure 1a,d) of UiO-PMAA-*b*-PMMA and UiO-NH_2_-PMAA-*b*-PMMA NPs covered the surface of the α-alumina membranes in a homogeneous manner (Figure 1b,e). This layer could thus serve as the preferential growing site for the secondary growth of the UiO particles on the membrane. Given the extremely thin deposited-seed layer it was not easy to measure the thickness. However, if only a single layer of UiO-polymer hybrid NPs were deposited, the thickness could be estimated from the diameter of UiO-polymer hybrid NPs (around 100 to 200 nm). In addition, the SEM cross-section images (Figure 1c,f) showed that a very little amount of NPs were deposited on the α-alumina support in comparison to the α-alumina support surface.

### 3.3. Secondary Growth

After the seeding of the support surface with the MOF-polymer hybrid NPs, a secondary growth step was applied following a solvothermal synthesis method. For that, the seeded α-alumina tubular membrane support was placed vertically in a cylindrical glass pressure vessel which contained a precursor solution following the recommended ration of Zr^4+^/BDC/H_2_O/DMF = 1:1:1:500 [15]. This low ratio of the precursors (ZrCl_4_ and terephthalic acid) was chosen because it favors the growth of interconnected crystals morphology.

**Figure 2:**
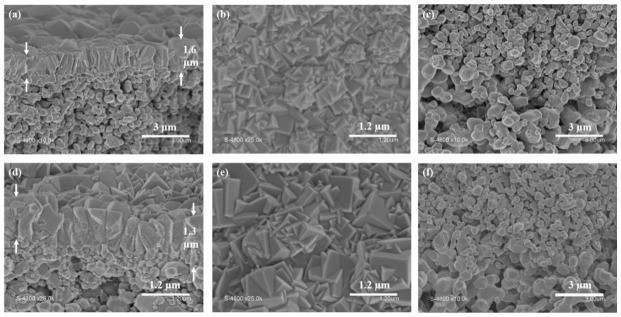
SEM images of (**a**) inner support layer cross-section, (**b**) inner support surface top view, (**c**) cross-section of inner and intermediate support layer with UiO-66 membrane; (**d**) cross-section of inner support layer, (**e**) top view of inner support surface, (**f**) cross-section of inner and intermediate support layer with UiO-66-NH_2_ membrane.

The SEM images of the polycrystalline membrane cross-sections, obtained after the secondary growth, showed the formation of compact, thin layers of UiO-66 and UiO-66-NH_2_ (Figure 2a,d), grown from the deposited UiO-PMAA-*b*-PMMA or UiO-NH_2_-PMAA-*b*-PMMA NPs. The average thickness of the membranes was approximately 1.6 µm (Figure 2a) and 1.3 µm (Figure 2d) for the UiO-66 membrane and UiO-66-NH_2_, respectively. The different thicknesses of the UiO-66 and UiO-66-NH_2_ secondary growth layer may be related to the slightly different crystallization process due to the presence of amine groups on the UiO-66-NH_2_ linker. It should be noted that these membranes are much thinner than their analogues reported in the literature (3–5 µm) [26], [38]. By examining, the top view in the SEM images (Figure 2b,e), no UiO-PMAA-*b*-PMMA or UiO-NH_2_-PMAA-*b*-PMMA NPs could be seen, suggesting that the inner surface of the α-alumina support was fully covered with a compact layer of inter-grown UiO-66 or UiO-66-NH_2_ crystals. Only a few UiO-66 or UiO-66-NH_2_ crystals (after the secondary growth step) could be observed by SEM (Figure 2c,f) inside the α-alumina support cross-section, confirming that the porosity of the α-alumina support layer was not altered. The fact that membrane growth occurs only on the surface of the α-alumina support confirms a successful seeding by both UiO-PMAA-*b*-PMMA and UiO-NH_2_-PMAA-*b*-PMMA NPs, ensuring the controlled secondary growth, towards the formation of a thin compact UiO-based membrane.

**Figure 3:**
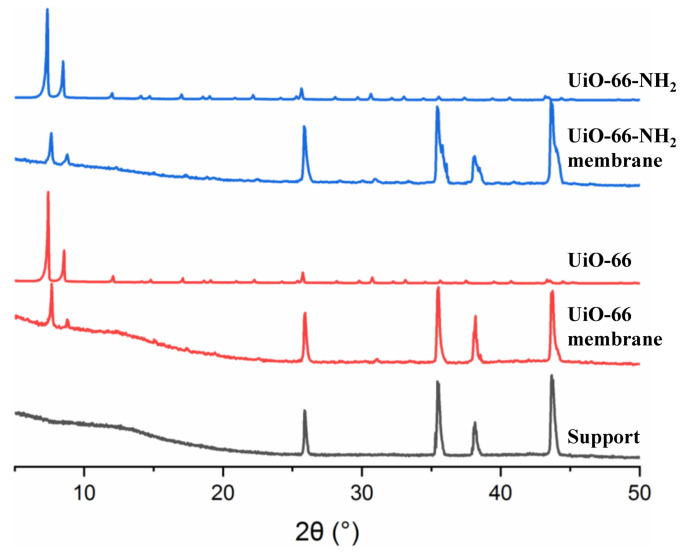
Experimental PXRD patterns of α-alumina membrane support, UiO-66 MOF-based membrane and polycrystalline UiO-66, UiO-66-NH_2_ MOF-based membrane and polycrystalline UiO-66-NH_2_.

The experimental PXRD patterns (Figure 3) confirmed the crystallinity of the as-synthetized UiO-66 and UiO-66-NH_2_ layers. Signals between 25° and 45° correspond to the α-alumina support. Two first diffraction peaks at 7.65° and 8.78°confirm the formation of crystalline UiO-66 and UiO-66-NH_2_. It can be seen that the patterns are sharp but less intense compared to the polycrystalline samples and some of the peaks are missing. This observation could be explained either by the support geometry (tedious analysis of non-flat supports), or the small quantity of UiO-66 or UiO-66-NH_2_ present on the membrane support (only few micrometers thick layer of deposited MOF). Moreover, the α-alumina support showed intense diffraction signals that could mask the UiO signals.

### 3.4. Single Gas Permeation Test

The as-prepared MOF membranes were characterized by single gas permeation measurements in order to check the quality of the membrane layers and their performance (membrane compaction and gas selectivity). Four pure gases (He, N_2_, CO_2_ and SF_6_) were performed at different transmembrane pressures (*ΔP*) from 0.25 up to 1 bar. Such a series of measurements allowed the mapping of the evolution of the gas permeability versus the transmembrane pressure and the determination of the ideal selectivity of the selected gas pairs.

**Figure 4:**
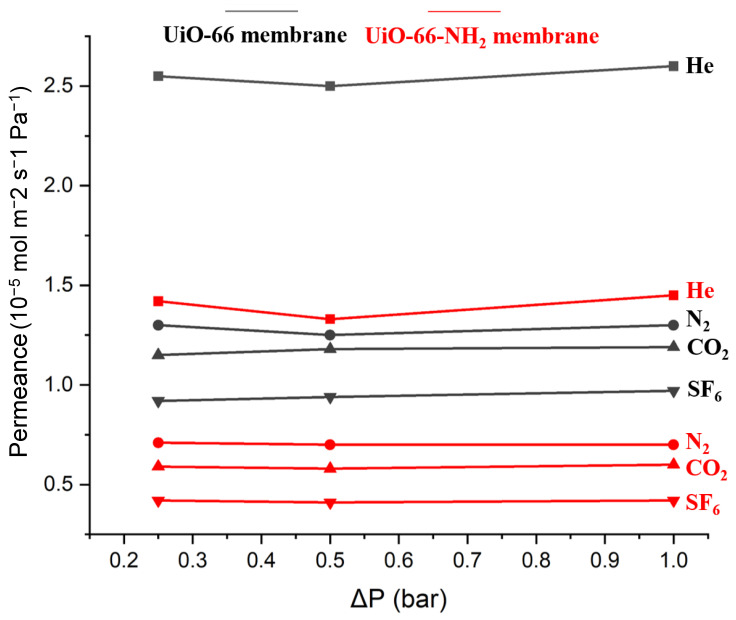
Single gas permeance (10^−5^ mol m^−2^ s^−1^ Pa^−1^) of He, N_2_, CO_2_ and SF_6_ versus transmembrane pressure (*ΔP*) for UiO-66 MOF-based membrane and UiO-66-NH_2_ MOF-based membrane.

Both UiO-66 and UiO-66-NH_2_ membranes revealed a constant permeance at the work transmembrane pressures (Figure 4). This result confirmed the virtually zero contribution of viscous gas flow and thus an absence of any big macroporous defects in the membrane structure. Hence, the prepared UiO-66 and UiO-66-NH_2_ membranes could be considered as homogeneous and compact, covering the entire active surface area of the α-alumina support. A slight increase in helium permeance as a function of transmembrane pressure could be attributed to the error associated with the measurements at high gas fluxes. When comparing the existing UiO-66 [22] and UiO-66-NH_2_ membranes [27], it was observed that the permeance of membranes prepared in this work were significantly higher. This could be most likely due to the extremely thin UiO-66 and UiO-66-NH_2_ layer, exhibiting lower hydraulic resistance during the gas permeation.

In order to evaluate the membrane separation properties, ideal selectivity for selected gas pairs were calculated (Table 1). When evaluating the ideal selectivity measured for the tested membranes it was noticed that they were always below the Knudsen selectivity values. This finding could be attributed to the presence of the inter-crystalline defects slightly above the mesoporous size range (>50 nm). These defects may result from insufficient crystal compactness after secondary growth and/or possible presence of defects in the UiO crystalline structure. The ideal selectivity of UiO-66-NH_2_ membrane was rather higher than of the UiO-66 analogue, which is most likely due to the better inter-grown UiO-66-NH_2_ crystals on the α-alumina support. From the results presented above, it is evident that further optimization of the membrane synthesis is necessary, with the aim of preparing more selective membranes with better sieving properties. Such optimization could be achieved by improving the secondary membrane growth protocol, e.g., by adding a modulator or changing the reaction conditions (time, concentration of UiO precursors, etc.).

### 3.5. Nanofiltration

**Figure 5:**
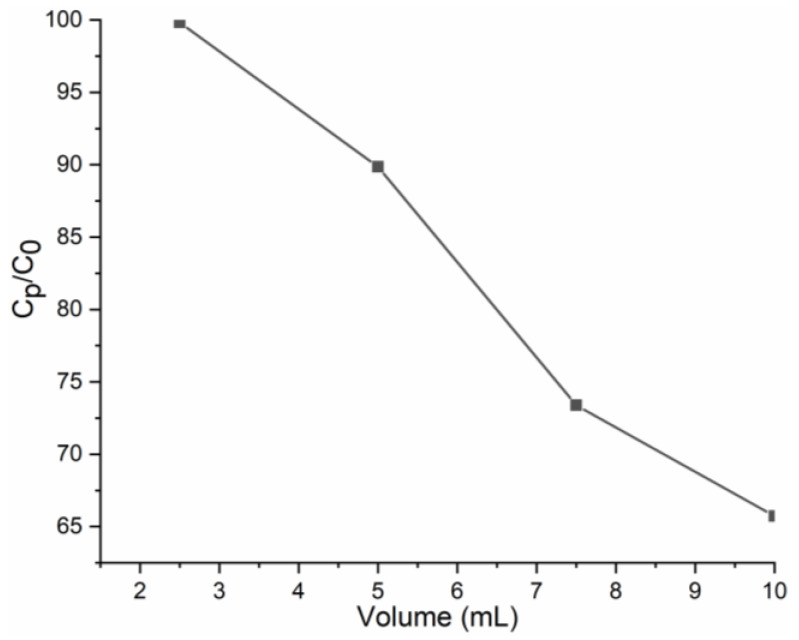
RhB rejection evaluation versus filtration volume through UiO-66-NH_2_ secondary growth membrane.

The membrane prepared with UiO-66-NH_2_ secondary growth with the best gas selectivity results obtained from the single gas permeation measurements was used for the removal of RhB from an aqueous solution. The membranes were conditioned at a higher pressure as compared to the filtration working pressure prior to dye filtration. This step ensures that the membrane thickness would be compressed to a stable level. As can be seen in Figure 5, as the filtration volume increases, the RhB rejection that occurs is greater. The filtration was carried out using a feed solution of 0.03 mM RhB (10 mL of filtrate was collected for 12 min). The average flux value was ca. 7.6 L m^−2^ h^−1^ bar^−1^ (Figure S6a). The decreasing flux during filtration indicated the adsorption of the RhB in the pores. The first fraction collected was colorless, with average UV absorbance of 0.036. This value translates to an almost 100% rejection of RhB. In the second fraction, the RhB rejection decreased to 90%. This decreasing trend was more prominent in the third and fourth fractions (73% and 66%, respectively). The calculated average RhB rejection was about 82%, suggesting an adequate filtration ability as compared to performant membranes used for separation of dyes. To be noted that the dye separation happened through the 1 µm of secondary growth layer which represented a quantity only 0.3 to 0.5 wt.% (about 30 mg) of the whole membrane module. To some extent, this limited the dye separation ability under continuous flow conditions. A closer look at the results suggested that the mechanism of the rejection was mainly due to the adsorption of RhB in the pores of the UiO-66-NH_2_ via acid–base interaction. After filtration, the membrane was soaked in 5 mL of water for 12 h (repeated once) and then was soaked in 5 mL of ethanol for 24 h. It was observed that the pink color was removed from the membrane and the water/ethanol had become colored; thus, indicating that the removed dye could be washed off from the membrane. Potentially, both the membrane and the separated dye could be recycled. However, this requires further studies.

## 4. Conclusions

In this work, a simple and innovative procedure is reported for the preparation of UiO-66 and UiO-66-NH_2_ membranes via a direct seeding of UiO-polymer hybrid colloidally stable NPs followed by the secondary growth of UiO-66 or UiO-66-NH_2_. Unlike other reported reactive seeding or polymer-assisted seeding methods, there was no need for the precursor immobilization or any extra steps to mix the MOF crystals with the polymer. Thus, the synthesized UiO-polymer hybrid NPs were stable in a colloidal solution and could be easily deposited on the support via dip-coating, resulting in the formation of a thin and homogenous seed layer.

This direct seeding of MOF-polymer colloidally stable NPs could be extended to other carboxylic acid-based MOFs for seeding. The new reported method allows facile seeding on different types of supports (polymer and alumina, flat sheets as well as tubular), potentially finding direct application in industry since they would be able to replace conventional filtration systems. The as-seeded supports were then subjected to classical solvothermal secondary MOF growth method, leading to UiO-based crystalline and compact membranes with interconnected UiO-66 and UiO-66-NH_2_ crystals. The gas permeance tests confirmed a high membrane permeability but low selectivity towards the four tested gases (He, N_2_, CO_2_, and SF_6_) suggesting the presence of some inter-crystalline defects in the membrane structure. Considering the membrane pore size, it is expected to be an attractive candidate in the fields of liquid separation (water or organic media). The RhB filtration corroborated the membrane stability and showed that the membrane has a good dye selectivity with a relatively high permeability. The membrane quality needs to be further improved by optimizing the secondary growth protocol to reach the level of molecular sieving. The data reported here, along with our previously reported work [32], highlight that the novel colloidally stable polymer-MOF NPs could be used as a seeding layer for growing MOF crystals of different types. In addition, this simple approach could easily be adapted to different substrates under different conditions, confirming their power in revolutionizing the membrane preparation and separation science. To further improve the viability of this method we need to find a way of performing the secondary growth step on several different (at least three) membranes in one pot to obtain membranes with identical structure. Currently, we are working towards this goal, making membranes from the presented material, demonstrating better separation, performance, reproducibility and reusability.

## Figures and Tables

**Table 1:** Ideal selectivity factor of different gas through the membranes (under *ΔP* = 1 bar).

	Membrane	UiO-66	UiO-66-NH_2_	Knudsen Selectivity Values
Gas				
He/N_2_		2.00	2.09	2.65
He/CO_2_		2.17	2.41	3.32
He/SF_6_		2.68	3.43	6.08
N_2_/CO_2_		1.01	1.15	1.25
N_2_/SF_6_		1.34	1.64	2.82
CO_2_/SF_6_		1.24	1.42	1.83

## Data Availability

The data presented in this study are available on request from the corresponding.

## Supplementary Materials

The following supporting information can be downloaded at: https://www.mdpi.com/article/10.3390/membranes13010065/s1, Figure S1: SEM images for (a,b) cross-section and (c) top view of α-alumina tubular membrane supports; Table S1: Experimental parameters for the synthesis of UiO-PMAA-*b*-PMMA NPs and UiO-NH_2_-PMAA-*b*-PMMA NPs; Figure S2: (a) TEM and (b) SEM images of UiO-NH_2_-PMAA-*b*-PMMA NPs; Figure S3: PXRD patterns of UiO-NH_2_-PMAA-*b*-PMMA powder; Figure S4: FT-IR spectrum for UiO-NH_2_-PMAA-*b*-PMMA powder (red) and PMAA-*b*-PMMA; Figure S5: N_2_ adsorption isotherms measured at 77 K for UiO-NH_2_-PMAA-*b*-PMMA powder; Figure S6: (a) Filtration flux of RhB solution versus filtration volume through UiO-66-NH_2_ secondary growth membrane, (b) calibration line of UV absorbance at 554 nm versus RhB concentration, (c) UV absorbance of different fractions. Reference [39] was cited in Supplementary Materials.
