# Supplementary material for: A Straightforward Method to Prepare MOF-Based Membranes via Direct Seeding of MOF-Polymer Hybrid Nanoparticles
Source: Membranes (Basel). 2023 Jan 4;13(1):65. doi: 10.3390/membranes13010065 (PMC9864354; doi:10.3390/membranes13010065)
Supplement: Supplementary file 1 [file membranes-13-00065-s001.zip › membranes-2089367-supplementary.pdf]

membranes

Supplementary Materials

A Straightforward Method to Prepare MOF-Based Membranes Via Direct Seeding of MOF-Polymer Hybrid Nanoparticles

Mingyuan Fang [1], Martin Drobek [1], Didier Cot [1], Carmen Montoro [2,*] and Mona Semsarilar [1,*]

[1] Institut Européen des Membranes, IEM UMR 5635, University of Montpellier, CNRS, ENSCM, 34095 Montpellier, France

[2] Inorganic Chemistry Department, Universidad Autónoma de Madrid, 28049 Madrid, Spain

* Correspondence: carmen.montoro@uam.es (C.M.); mona.semsarilar@umontpellier.fr (M.S.)

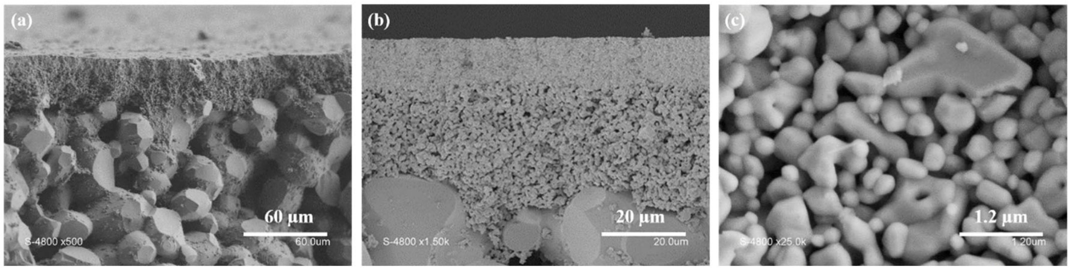

Figure S1. SEM images for (**a**,**b**) cross-section and (**c**) top view of α-alumina tubular membrane supports.

Table S1. Experimental parameters for the synthesis of UiO-PMAA-*b*-PMMA NPs and UiO-NH_2-PMAA-*b*-PMMA NPs.

NPs	$ZrCl_4$ (mmol)	Organic Linker (mmol)	PMAA-*b*-PMMA (mmol)[a]	PMAA-*b*-PMMA 20 wt% in EtOH (mg)[b]
UiO-PMAA-*b*-PMMA	0.5	0.5	3.2×10^{-3}	290
UiO- NH_2-PMAA-*b*-PMMA	0.5	0.5	3.2×10^{-3}	290

[a] One polymer chain of PMAA-*b*-PMMA containing 64 units of carboxylic functions.[b] Average molecular weight of PMAA-*b*-PMMA is calculated from $PMAA_{64}$-*b*-$PMMA_{124}$.

Characterization results for UiO-NH_2-PMAA-*b*-PMMA NPs

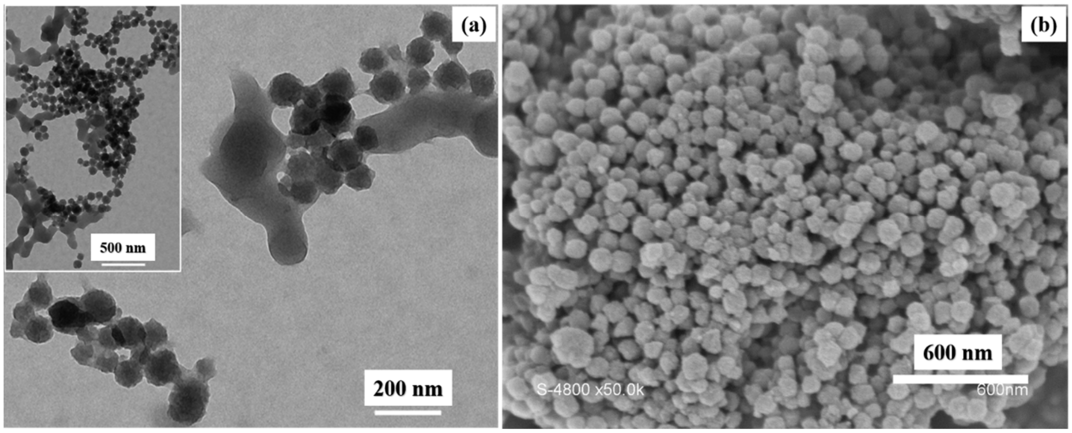

Figure S2. (**a**) TEM and (**b**) SEM images of UiO-NH_2-PMAA-*b*-PMMA NPs.

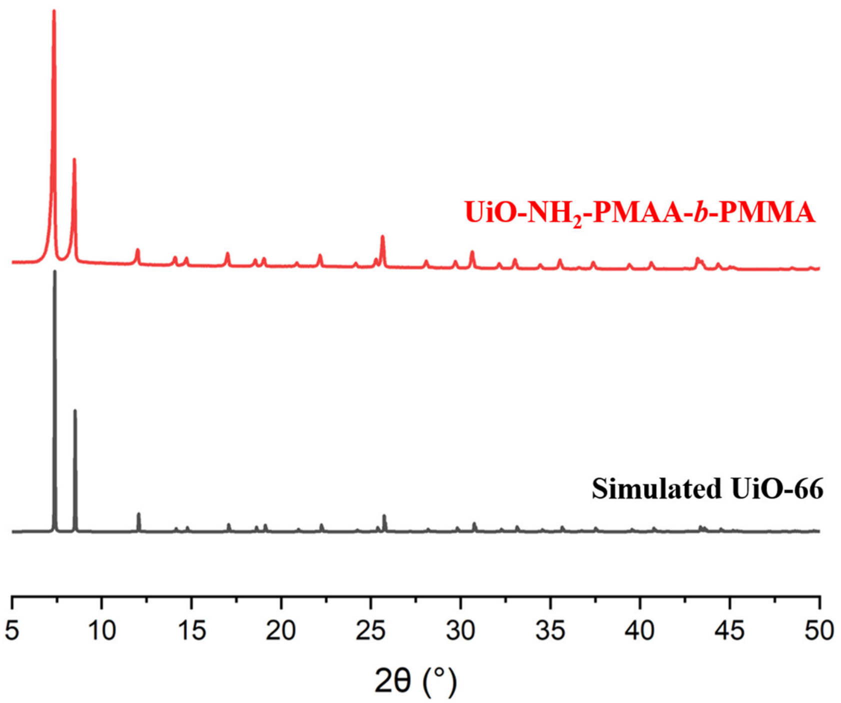

Figure S3. XRD patterns of UiO-NH_2-PMAA-*b*-PMMA powder (red) and simulated UiO-66 patterns (black) [1].

Powder XRD (Figure S3) of UiO-NH_2-PMAA-*b*-PMMA pattern show first two main peaks at 7.40° and 8.55° (2θ) charactering the formation of UiO-66 structure. The diffraction peaks are sharp and intense indicating that the presence of PMAA-*b*-PMMA NPs does not affect the crystalline phase growth of the UiO-66-NH_2.

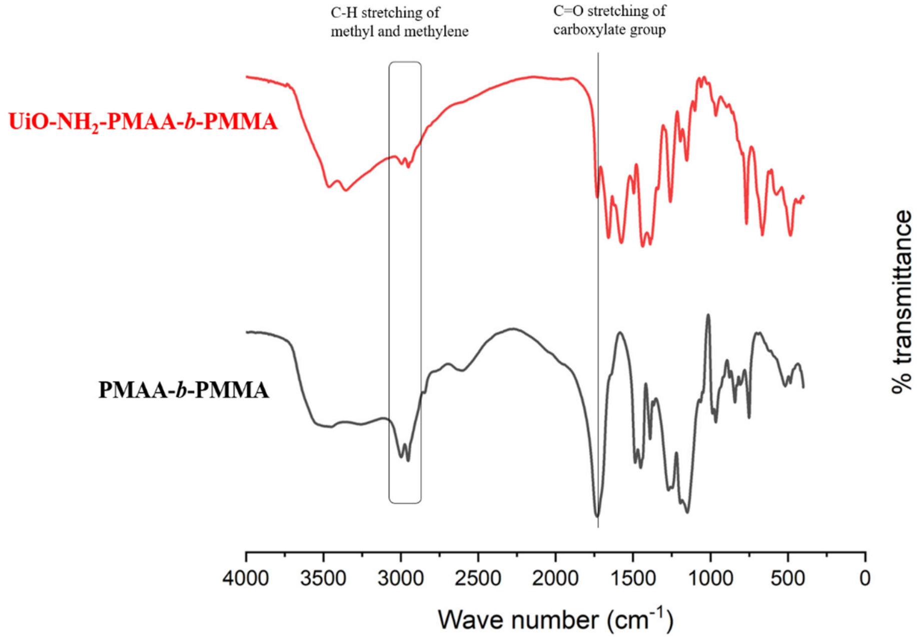

Figure S4. FT-IR spectrum for UiO-NH_2-PMAA-*b*-PMMA powder (red) and PMAA-*b*-PMMA (black).

The UiO-NH_2-PMAA-*b*-PMMA powder was further analyzed by FT-IR measurements (Figure S4). The broad and intense band between 3500 and 3300 cm^{-1} is related to the presence of N-H stretching of the amine group. C-H stretching of methyl and methylene groups between 2995 and 2955 cm^{-1} and an intense C=O stretching band of carboxylate group at 1730 cm^{-1} can be found. These signals prove that the PMAA-*b*-PMMA NPs were incorporated in UiO-NH_2-PMAA-*b*-PMMA samples.

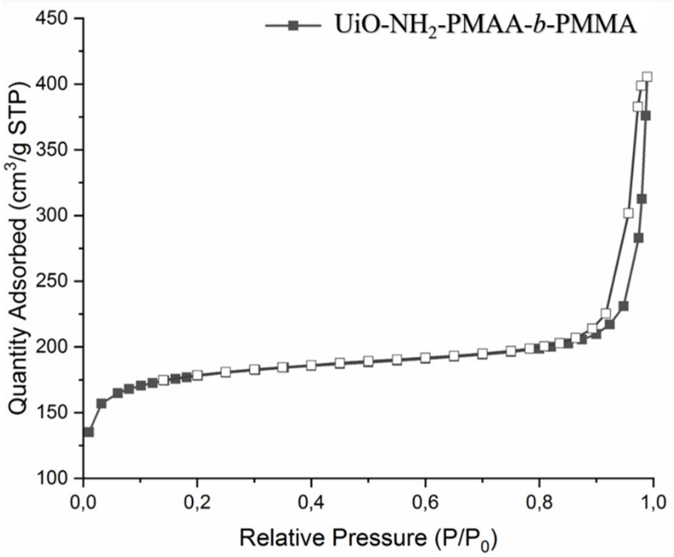

Figure S5. N_2 adsorption isotherms measured at 77 K for UiO-NH_2-PMAA-*b*-PMMA powder. Filled and empty symbols represent adsorption and desorption, respectively.

Nitrogen adsorption isotherms (Figure S5) of the UiO-NH_2-PMAA-*b*-PMMA powder, exhibited a mixture of type I and IV isotherm at 77 K with a Brunauer–Emmett–Teller (BET) surface area of 605 $m^2\ g^{-1}$ indicating the existence of microporous and mesoporous structure of UiO-NH_2-PMAA-*b*-PMMA NPs. This also corroborated that the presence of the polymer NPs did not affect the pore accessibility of the UiO-NH_2-PMAA-*b*-PMMA structure.

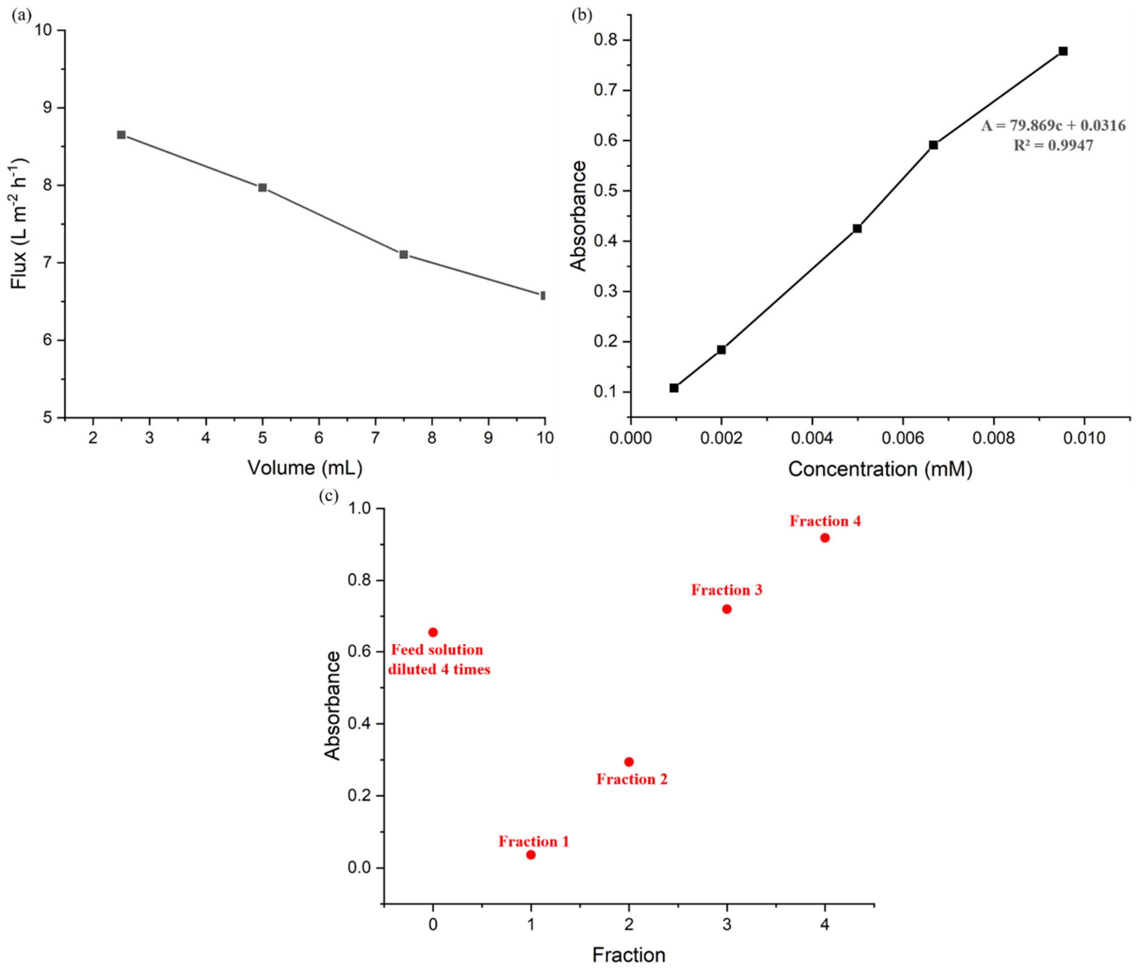

Figure S6. (**a**) Filtration flux of RhB solution versus filtration volume through UiO-66-NH_2 secondary growth membrane, (**b**) calibration line of UV absorbance at 554 nm versus RhB concentration, (**c**) UV absorbance of different fractions.

Reference

1. Øien, S.; Wragg, D.; Reinsch, H.; Svelle, S.; Bordiga, S.; Lamberti, C.; Lillerud, K.P. Detailed structure analysis of atomic positions and defects in zirconium metal-organic frameworks. *Cryst. Growth Des.* **2014**, *14*, 5370–5372, doi:10.1021/cg501386j.

Disclaimer/Publisher's Note: The statements, opinions and data contained in all publications are solely those of the individual author(s) and contributor(s) and not of MDPI and/or the editor(s). MDPI and/or the editor(s) disclaim responsibility for any injury to people or property resulting from any ideas, methods, instructions or products referred to in the content.
